# Understanding Care Navigation by Older Adults With Multimorbidity: Mixed-Methods Study Using Social Network and Framework Analyses

**DOI:** 10.2196/11054

**Published:** 2018-11-14

**Authors:** Jolien Vos, Kathrin Gerling, Conor Linehan, Aloysius N Siriwardena, Karen Windle

**Affiliations:** 1 UCL Interaction Centre University College London London United Kingdom; 2 Group T Leuven Campus Technology Cluster Computer Science Technology Katholieke Universiteit Leuven Leuven Belgium; 3 School of Applied Psychology University College Cork Cork Ireland; 4 Community and Health Research Unit School of Health and Social Care University of Lincoln Lincoln United Kingdom; 5 NatCen London United Kingdom

**Keywords:** care navigation, long-term conditions, multimorbidity, older adults, social network analysis

## Abstract

**Background:**

Health and social care systems were designed to be used primarily by people with single and acute diseases. However, a growing number of older adults are diagnosed with multiple long-term conditions (LTCs). The process of navigating the intricacies of health and social care systems to receive appropriate care presents significant challenges for older people living with multiple LTCs, which in turn can negatively influence their well-being and quality of life.

**Objective:**

The long-term goal of this work is to design technology to assist people with LTCs in navigating health and social care systems. To do so, we must first understand how older people living with LTCs currently engage with and navigate their care networks. No published research describes and analyses the structure of formal and informal care networks of older adults with multiple LTCs, the frequency of interactions with each type of care service, and the problems that typically arise in these interactions.

**Methods:**

We conducted a mixed-methods study and recruited 62 participants aged ≥55 years who were living in England, had ≥2 LTCs, and had completed a social network analysis questionnaire. Semistructured interviews were conducted with roughly a 10% subsample of the questionnaire sample: 4 women and 3 men. On average, interviewees aged 70 years and had 4 LTCs.

**Results:**

Personal care networks were complex and adapted to each individual. The task of building and subsequently navigating one’s personal care network rested mainly on patients’ shoulders. It was frequently the patients’ task to bridge and connect the different parts of the system. The major factor leading to a satisfying navigation experience was found to be patients’ assertive, determined, and proactive approaches. Furthermore, smooth communication and interaction between different parts of the care system led to more satisfying navigation experiences.

**Conclusions:**

Technology to support care navigation for older adults with multiple LTCs needs to support patients in managing complex health and social care systems by effectively integrating the management of multiple conditions and facilitating communication among multiple stakeholders, while also offering flexibility to adapt to individual situations. Quality of care seems to be dependent on the determination and ability of patients. Those with less determination and fewer organization skills experience worse care. Thus, technology must aim to fulfill these coordination functions to ensure care is equitable across those who need it.

## Introduction

### Background

While people now live longer than previous generations, they do not necessarily live well for longer [1]. With the increase in life expectancy, there is also an increase in long-term conditions (LTCs), such as arthritis, diabetes, and heart disease. In addition, a growing number of older adults are diagnosed with ≥2 LTCs, also referred to as multimorbidity [2,3]. Health and social care systems were primarily designed for the management of people with single diseases and acute conditions rather than those with multiple LTCs, resulting in difficulties in care provision and navigation (ie, finding the right type of care, in the right place, and at the right time) for those with multimorbidity [4]. Care systems are, for example, often not connected in the way patients expect [5,6]. This can lead to expectations remaining unmet, as well as over, under, and inappropriate use of the care system [5,7,8]. Furthermore, the incurable nature of LTCs, combined with the burden they can place on people’s lives, increases the importance of maintaining and improving the quality of life [4]. Patients with multimorbidity especially value clear communication and accessibility of providers. Particularly for older individuals with multimorbidity, there is an urgent need for support in appropriately navigating the care system to maximize health and well-being.

### Prior Work

One approach to addressing this need is the provision of designated “care navigators”—professionals who support patients in their “pathway” or “journey” through the care system. In their task of guiding patients through the system, care navigators focus on the needs of individuals. Studies in the cancer care setting have shown the benefits of care navigators [9-11]. However, despite having a positive effect on patients’ satisfaction, quality of life, and functionality [7,12], the high cost of care navigators remains a barrier to their wider employment [13]. Furthermore, their involvement in the patients’ journey tends to be limited to short amounts of time [14] and focus on single LTCs (eg, cancer) instead of multiple LTCs [6].

A potentially more cost-effective, accessible, and equitable solution lies in the use of technology to aid care navigation [8,15]. Indeed, it could be argued that care navigation is an information management and communication problem; these are exactly the types of problems that information and communications technology is well suited to solving. Some work is beginning to emerge on this topic. For example, Yao et al [15] proposed the design of a navigation support system for patients modeled on decision-support tools more commonly designed for clinicians. By providing patients with a unified and integrated view of their specific care continuum, Yao et al [15] aimed to help patients understand and manage their health care. Their prototype design did not directly involve patient data or consultancy but focused on pathways derived from medical guidelines. Yao et al [15] commented that navigation programs need to truly focus on patients to help them to manage this task, suggesting that a better understanding of patients’ needs concerning care navigation and multimorbidity is required to design effective support systems. Zulman et al [14] addressed this issue by outlining patients’ need. The following 3 themes emerged from their study: (1) *patients with multimorbidity manage a high volume of information, visits, and self-care tasks*; (2) *they need to coordinate, synthesise and reconcile information from multiple providers and about different conditions*; and (3) *their unique position at the hub of multiple health issues requires self-advocacy and expertise* [14].

However, Zulman et al [14] did not provide a detailed understanding of stakeholders involved in the care network. To date, no previous study has investigated the structure of the care network of older people with multiple LTCs. There is very limited knowledge available to researchers on how older people with multimorbidity interact and engage with their care network. These gaps in knowledge make it difficult for anyone to design appropriate care navigation support for these patients.

### Goal of This Study

For technology to support older adults in care navigation, an understanding of both the care system and people’s experiences of that system is needed. This study is the first step in the experience-centered design [16] of tools to support care navigation. The goal of this paper is to describe and analyze the challenges inherent to care navigation, and in doing so, outline design opportunities for technology to support older adults with multimorbidity when navigating the care system. As such, we contribute to the current knowledge by providing a systematic exploration of older people’s existing experiences, needs, and goals in care navigation, while relating these to their personal care network (PCN; people providing them with care). Using a mixed-methods approach, this study aimed to identify the type and number of caregivers (formal and informal) involved in the care of older people with multimorbidity. Through quantitative [social network analysis (SNA)] and qualitative (framework analysis) methods, we examine and explore older people’s experiences and needs in relation to navigating their care. This analysis considers the breadth and depth of participants’ experiences but still allows actionable reflections on challenges and opportunities for the human-computer interaction community when designing for multiple conditions.

## Methods

### Study Design

We performed a pragmatic mixed-methods study to understand care navigation from the perspective of older adults with multiple LTCs (refer [10,17] for a detailed discussion of mixed-method study designs). The intention was 2-fold as follows:

1. Understanding PCNs surrounding older people with multimorbidity. *This included identifying which caregivers were involved, why and how were they involved.*
2. Understanding the experiences of older people with multimorbidity in relation to care navigation. *This encompassed examining how the PCN currently functioned and how it should be functioning for older people with multimorbidity*.

Quantitative (questionnaire) data were needed to help answer the question of “who” was involved in the PCN of a wide range of participants, and to some extent, “why” they were involved. Qualitative (semistructured interview) data were used to give in-depth information about the latter, as well as details of “how” those people were involved, and in “what” way the network functioned. A tranche of quantitative data was initially collected and analyzed. This initial analysis was used to guide the design of the qualitative interviews. Specifically, interviews focused on topics that were recognized as important in the initial analysis. Interviews were then started, with the remainder of the quantitative data being collected and analyzed concurrently with the interview strand. Ethical approval for the research was obtained through the “University of Lincoln’s” ethics board and the Ethical Committee of the “national health care body.”

### Sample and Recruitment

Eligible participants had to be aged ≥55 years, living in England, and diagnosed with at least 2 LTCs. We aimed for a minimum of 50 questionnaire respondents and a 10% subsample for the interviews. The study was advertised through a number of methods. First, emails and social media messages were posted by both a university and an age-related nongovernmental organization. Second, flyers were created and placed, with agreement, in churches, community halls, and charity shops. Third, posters and information sheets were placed in 101 general practices. Fourth, people engaging with a pilot care navigation project run by a nongovernmental organization were contacted directly. Once the questionnaire was completed, eligible respondents (ie, those living locally to the lead researcher) were offered the option to participate further through a semistructured interview. Those who decided to do so, were contacted to further discuss the study, check their consent, and clarify any further questions. In agreement with the participants, a place for the interview was decided (usually the participants’ home).

### Data Collection

To understand the range of experiences encountered by people with multimorbidity in navigating the health and social care system, it was necessary to capture information about participants’ communications, interactions, and relationships with a range of different people, services, and institutions involved in their care. Two distinct instruments were designed to collect the data in this mixed-methods study.

#### Social Network Questionnaire

**Figure 1:**
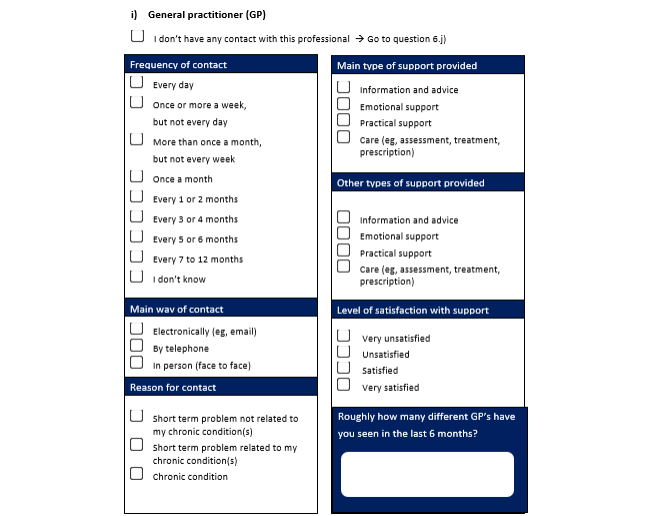
An example question of the social network questionnaire for the study.

Social network questionnaires have been found to be useful for the assessment of connections and relationships between people or social actors [18]. The “egocentric” SNA is a subtype of SNA that aims to specifically understand the relationships surrounding one focal unit or actor in a network [18]. The egocentric SNA provided a method for us to assess the patients’ perspective of their own care network. Very few examples of validated and nonvalidated questionnaires for social network data were found at the start of this study [19,20], and none existed specifically to gather data about a participants’ PCN. Therefore, a new questionnaire was developed. Our questionnaire was designed primarily on “name generator” questions. These questions asked participants which formal and informal caregivers they were in contact with, the frequency of contact, and the reason for contact (eg, treatment, support; Figure 1). The majority of questions were close-ended, allowing direct comparison of the data across participants (Figure 1).

An initial draft of the questionnaire was reviewed by 6 members of a Patient and Public Involvement group. Based on their feedback, an adjusted version was sent out for pilot-testing among 3 members of the public (who met the inclusion criteria for the study), 2 members of a “Later Life” forum, and 2 academics independent from and unfamiliar with the research. This group of people completed the questionnaire and provided feedback that led to final adjustments of the questionnaire (Multimedia Appendix 1).

#### Semistructured Interviews

The questionnaire was not intended to provide nuanced data on, for example, the “strength” of connections that patients had with care providers; to address this, semistructured interviews were planned (Multimedia Appendix 2). The final topic guide included questions on patients’ needs regarding (digital) care navigation support and their current experiences and barriers to using such technology.

### Data Analysis

This study gathered both quantitative and qualitative data, thus requiring a number of different types of analyses, plus a strategy for integrating data across those methods.

#### Quantitative Analysis: Social Network Analysis and Descriptive Analysis

**Figure 2:**
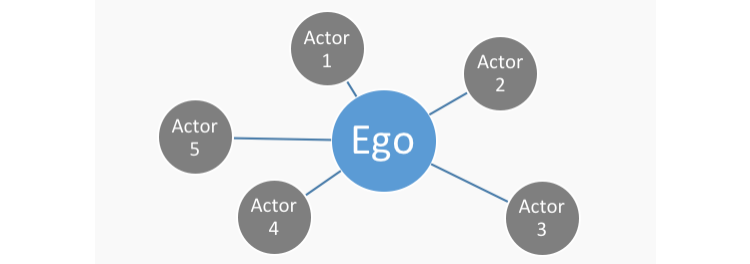
The example network graph displaying nodes and edges.

This study used the SNA to interpret questionnaire responses. In care settings, SNA has, for example, been used to describe and understand the social aspects of communication patterns [21], investigate the impact of social capital on health and well-being [22], and look at the influence of social networks on frail older people’s life satisfaction [23]. The SNA includes 2 main components—“actors” and “relationships.” Actors in the SNA are represented by points and referred to as nodes. Nodes are the individual units that are connected by the relations (ties). The ties (relationships) or “edges” in the SNA are represented by lines (Figure 2) and can display any possible connection between the nodes of interest such as friendships, collaborations, and information flows.

To visually support the analyses of these structures, the SNA uses graphs, also called sociograms [24], an example of which is shown in the simplified example in Figure 2. We used SPSS Statistics V22 (IBM Corp) for *descriptive analysis* of the data and Gephi 0.9.1 [25] as *visualization and exploration* software assisting the SNA and providing sociograms of the PCNs.

#### Qualitative Analysis: Framework Analysis

Framework analysis [26] was selected as the most suitable method to analyze the semistructured interviews. Framework analysis is a specific type of thematic analysis, usually with greater emphasis on the transparency of the analytical process [26] and linkage between the stages of analysis [27]. This analysis was an inductive, iterative, and continuous process in this study. It allowed for concepts to emerge as we progressed through the analysis process. However, at the same time, we had a clear understanding of the purpose of the research and the question that needed answering [28].

The qualitative analysis involved 3 separate stages, although these were not necessarily linear in progression and moving between different stages was not uncommon. In the first instance, the transcripts were line-by-line coded. This process was assisted by the NVivo 10 software package and resulted in several open codes (eg, difficulties finding the “right” person to contact and perception of limited communication between providers) that were later grouped as categories (eg, barriers in care navigation) and supported the themes from the framework. Second, both within and between transcripts, a search was conducted for remarkable and noticeable differences and similarities between participants (eg, patients with family living nearby vs those with family further away). Third, reoccurring codes, differences, and similarities were grouped and brought together as subcategories within the following 7 themes: (1) meaning of the PCN; (2) structure of the PCN; (3) roles and responsibilities in the PCN; (4) first point of contact; (5) service organization or operation; (6) PCN interaction and communication; and (7) technology.

## Results

### Sample Characteristics

We recruited 62 participants, all aged ≥55 years, living in England, and diagnosed with ≥2 LTCs. While 37 participants accessed the questionnaire via a Web-based link, 25 completed a paper version. Of all, 28 participants reported as male, 14 as female, and the remaining 20 preferred not to say or left the question blank. On average, questionnaire participants were 72 years old (range, 55-94 years). Participants indicated they had been diagnosed with a variety of LTCs, the five most common being musculoskeletal conditions, cardiovascular disease, bowel diseases, respiratory conditions, and diabetes. We excluded participants with conditions affecting cognitive and memory abilities from the study. No significant relationship was found between age and the number of LTCs (*r*=−.112, *P*=.51), and no significant difference was observed in the sample for the number of LTCs between men and women (*F*_*24*_=2.327, *t*_*24*_=−1.239, *P*=.23). All participants reported they had been diagnosed with their first LTC >2 years ago. The majority of participants who answered the question relating to the time of diagnosis (n=36) had their first diagnosis ≥10 years ago (52.8%, 19/36), and 47.2% (17/36) had the diagnosis <10 years ago.

Semistructured interviews were conducted with a rough 10% subsample of the questionnaire sample (4 women and 3 men). On average, interviewees aged 70 years (range, 57-83 years) and had 4 LTCs (range, 2-8).

### Understanding the Personal Care Network: Caregivers

**Figure 3:**
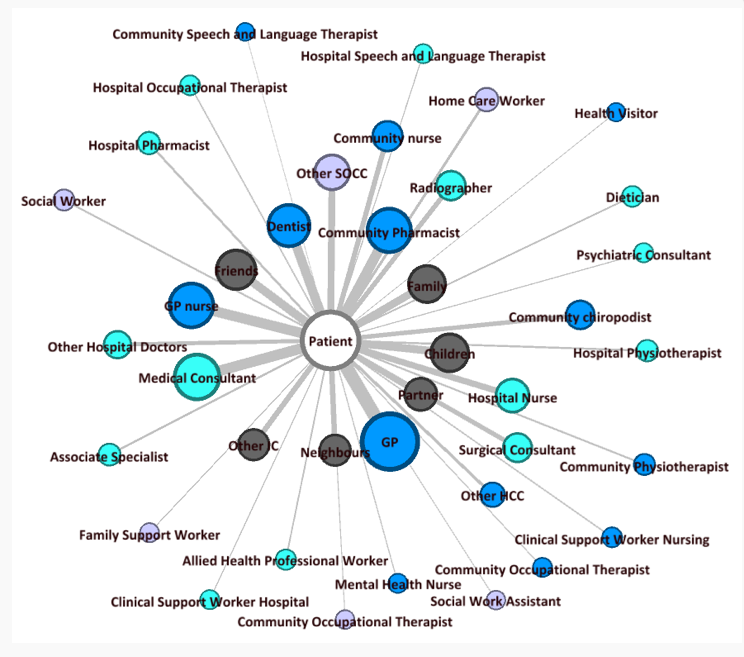
Actors involved in participants’ personal care networks. Colors represent separate domains of care: blue, health care actors in the community (HCC); cyan, health care actors at the hospital (HCH); purple, social care actors in the community (SOCC); gray, informal care (IC). GP: general practitioners.

To understand our participants’ PCNs, we identified, for each participant, which caregivers were involved, as well as their reason for involvement. Across the questionnaires, a total of 39 different actors were reported by participants (Figure 3). Actors closer to patients and conveying stronger ties (ie, thicker lines) were more frequently indicated by participants. Consequently, actors further away from and connected with patients through thinner ties were overall less indicated by the sample. The closeness or distance of these actors to patients is also represented by the size of the nodes. Bigger and smaller nodes, respectively, reflected actors more or less frequently mentioned to be involved in the PCN of participants. On an individual level, the number of *important* actors varied across participants, from as little as 1 to as many as 20. Regarding participants’ *contact* with actors, similar results were observed. On average, the PCN of patients contained 7 actors.

Those (2/7) who lived further away from their immediate family or did not have certain people within that group (eg, partner), tended to elaborate in greater detail the structure of those living around them. Interviewees who did not have their family nearby showed higher reliance on neighbors, friends, and even people in the wider community.

### Understanding the Personal Care Network: Domains of Care

We identified 4 domains of care as follows: health care actors in the community (HCC); health care actors at the hospital (HCH); social care actors in the community (SOCC); and informal care (IC) actors. Figure 3 displays the structure of the PCN according to these domains of care. The different domains of care were allocated different colors to provide a domain-sensitive graph. The average amount of actors indicated as important per domain was slightly higher for HCC (n=4) than the other domains (SOCC, 1; HCH, 3; and IC, 2). The domain-specific averages relating to contact did not show much internal variation; generally, participants indicated 3 HCC, HCH, and IC actors they were in contact with and 1 in the domain of SOCC.

Both the interview and questionnaire data suggested a smaller involvement of formal social care than any other type of care (ie, hospital care, primary care, and informal and third sector care). Less than a third (30.6%, 19/62) of the participants indicated ≥1 SOCC actors to be involved in their PCN. Over double this number (67.7%, 42/62) was reported for HCC actors, and 51.3% (32/62) indicated the involvement of HCH and IC actors.

### Understanding the Personal Care Network: Levels of Care

Unlike the questionnaire, the interviews did not predefine domains (ie, SOCC, HCC, HCH, and IC) for inquiry. As such, the groups of care that emerged from the interview data were based on patients’ perceptions of the type or levels of support they provided. In other words, this added detail on why certain actors were involved in patients’ care. When describing the PCN during the interviews, participants tended to distinguish 3 levels of support as follows: support provided on a *day-to-day* basis, frequently used services or providers for *monitoring and follow-up*, and *“exceptional” care* delivered by professionals.

[…] there are local charities, there’s the stoma nurses, there’s the local Ileostomy association. I go to see a consultant once a year at the hospital so to me that is the…my care network, as well as friends and family.

pp7

The interviews revealed that daily continuous support was mainly provided by informal caregivers, whereas follow-up activities and expert care were situated, respectively, on the level of primary and secondary care.

[…] So you’ve a group of more exceptional people to access than you have informal care givers who are there on a day to day basis. And then you’ve those that you basically access on a frequent basis to keep in check with the conditions that you have.

pp5

**Figure 4:**
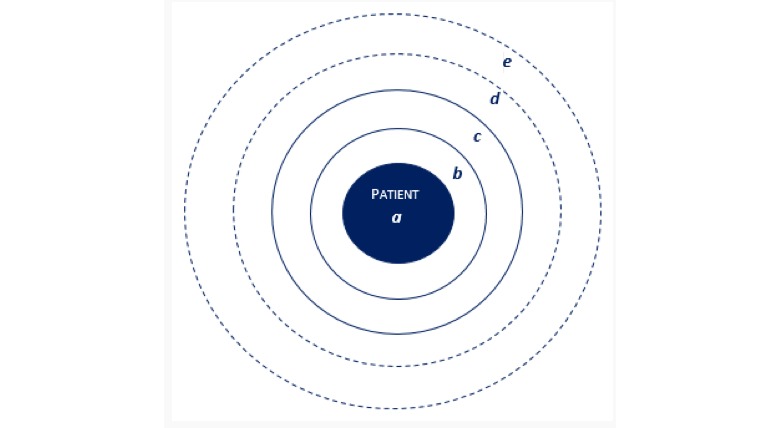
Representation of the 5 separate care actors identified from the personal care network analysis.

Integration of the data further led to the identification of *5 main categories of actors* in the PCN (Figure 4)—the patient himself or herself (1), the general practitioner (GP) practice (2), the informal network (3), the experts involved depending on the type of LTCs patients were diagnosed with (4), and additional services used as required (5). The first 3 (1-3) were found to be the “core” of the PCN, remaining relatively stable across patients’ time living with LTCs. The presence and number of experts (4) and additional services (5), however, were more subject to change.

### Patients’ Personal Care Network Experience

To help us understand patients’ experience in terms of care navigation, we examined the functioning of these 5 main categories of actors in the PCN. We investigated the functioning of patients (1); GP practice (2); informal network (3); experts (4); and additional services (5) in terms of their roles and responsibilities.

#### Patient: Self-Care, Disease Management and Assertive Communicator

The interviews showed a strong sense of awareness among participants in terms of their own responsibility as a patient. Interviewees (n=7) pointed out how their own actions contributed to their health (physically) and well-being (mentally). From the interviews, 2 distinct types of behavior emerged—actions undertaken to remain as healthy as possible (self-care) and measures taken to control and manage one’s LTCs (disease self-management).

Another element that emerged (3/7) was the need to find activities that were possible or adjustable to the interviewees’ LTCs. One participant, in particular, found this a struggle.

[…] go to the gym at least three times a week. And do euhm, we do aqua aerobics as that is all I can do, I can only do things in the pool. Because, because anything else is not good for arthritis. So yes, that’s mainly what we do to stay healthy and try to eat healthy.

pp2

Two patients disclosed a mental health issue (ie, depression). However, all interviewees spontaneously stated the importance of self-care in terms of mental health (well-being), sometimes (n=2/7) even if that meant potentially going over their physical limit.

[…] my responsibility is obviously to keep as healthy as possible, mentally and physically.

pp6

Self-care behavior also included seeking help from the actors in the PCN to, for example, prevent worsening of the situation.

In relation to disease self-management, interviewees emphasized their responsibility in terms of medication adherence, attendance of appointments, and daily monitoring of their conditions. Depending on LTCs participants were diagnosed with, disease self-management and self-care sometimes overlapped and at other times challenged one another.

Based on the experience, participants developed their own personal ways to practically manage their LTCs and the people involved in their PCN. The use of diaries to keep track of appointments was present in all interviewees (n=7). In addition, some (2/7) kept a log of the reason and outcome of appointments as well as their medication.

Diary and yeah I’ve various things on my computer, like I have a medication list knowing what the medication is for […] I have, every time I go to the GP or go to the doctor or go to the to the hospital, I’ve a list of every time I’ve been. Because often when you go they’ll say to you ‘and when did you last do this?’ and I was thinking I’ll never going to remember so I actually got a log, I started it in 2008, every appointment I’ve ever been to.

pp2

Occasionally, 2 of the 7 patients mentioned that they felt as if they were not given the tools to keep track of their health and/or care.

You know all this business with that they said they gonna let, you can access your medical record? But you can’t! [pp4].

The third main activity that arose across interviews was the patients’ need to be assertive, determined, and proactive. Patients felt that the way the care system was set up required them to persevere in their navigation and deal with a number of complex barriers in accessing services, which could add frustration. The process involved in dealing with switchboards or finding the “right” person to talk to was found challenging.

But if I hadn’t sort of kept phoning them I probably would have been just struggling on my own.

pp7

PP: It’s like a minefield.

I: Mhm, how do you do that (navigation)?

PP: With difficulty, with difficulty… you know, you spend hours on the phone, press button A, press button B, number one for this, number nine for that, five for this… and all the while everything is a recorded, recorded answer, it’s a program, everything is robotic, you don’t speak to a person. It’s a minefield, it’s a battlefield trying to get through, you speak to one, “oh I can’t deal with that I’ll put you through to my colleague” and you explain everything over again and then “oh no you need to speak to such and such” […] and then I get frustrated because they’ve given me the wrong number. And so if they’re doing their job properly, again like I say there’s a right way and a wrong way.

pp4

Sometimes patients were left at a “loose end”; not having anything or anyone in place to follow-up on the situation. At other times, patients felt they were sent “backward and forward” across the system.

[…] the other thing I expected from hospital and I kept asking for it. Is you know, some sort of physiotherapy type of thing […] I was hoping that somebody somewhere would you know suggest physiotherapy or something. But there was absolutely nothing, you feel and this is why I had the mental health problem to start off with […] And nobody was giving you any advice.

pp7

[…] You then get a phone call from somebody on the switchboard, who then passes it on to somebody else, euhm, to a manager, to see you then, to see that you… you speak to the telephone person who then puts you on to somebody for, I I thought they were from the team, the safeguarding team but no they were only a receptionist that takes the minor details, who then passes you on to somebody else who you speak to then for an hour on the phone, who then says I will pass your details on to a line manager to see if you were a, a visit from a social worker so you tell the story to five people… and then you might have forgotten something which happened in the first place or… you could have added a bit on, do you know what I mean?

pp4

#### General Practitioner Practice: Gatekeeper and General Monitor

GP practices were reported by participants, both in the interviews and questionnaires, to hold a central position in their PCNs. The interviews revealed that this central position was the result of and strengthened by 2 main roles—the GP’s “gatekeeper” role and their function as a general monitor of patients’ health.

All interviewees discussed the process of referral through GP practices, and the GP in particular. Access to different (health and social care) services in primary, secondary, and sometimes even third sectors care was gained through the GP. Exceptions to this were patients (partly) choosing to take the route of private care (2/7), but even then, the GP was often asked for information on services that could be approached.

Yes, everything has to go through the GP, well not the dentist, but everything else goes through the GP surgery.

pp2

I got in from our local general practitioner, a list of companies offering private auxiliary care help.

pp6

Apart from being the figure in charge of referral, providing access to other parts in the care system, the GP practice was also seen as the place to monitor patients’ general health. General check-ups were often scheduled ahead (eg, every 6 months) to keep an eye on patients’ LTCs such as diabetes. The disease-specific follow-up (if needed) did, however, not fall under the responsibility of the GP practice (see section on experts) according to the study participants.

#### Informal Network: Day-to-Day Support

Drawing on the interview analysis, the informal network was reported to be the main source for patients’ day-to-day support. Depending on its structure (ie, solely family and friends or also including the wider community), roles and responsibilities of informal actors were shared differently and divided among those involved.

[…] We are lucky at our bowls club because we have a restaurant and we have a bar, you know so it is very convenient. And this to me is that sort of care in the community is where people look out for each other you know?

pp1

Day-to-day support mainly involved practical and emotional support. Practical support, such as transportation, was often mentioned (n=5/7) when discussing the importance of family and friends.

Sometimes use a friend of church for attending the doctor at surgery when I haven’t been able to drive myself […] I have an address book and I try not to bother the people with surnames starting with ‘A’ too frequently (laughs). Which today we will stick the pin in the ‘W’s’ or the ‘S’s’ or the ‘C’s’ or...you know.

pp6

Second, friends (and sometimes the wider informal network) were a source of information. Information and advice were in particular sought in relation to “connections” friends might have access to and the patient (currently) did not.

It was noted that for advice on medical issues, participants were more inclined to rely on professionals than on informal actors.

I don’t wanna bother them with things they can’t necessarily answer. I mean if effectively it’s a medical problem you need to see a doctor, don’t you? You don’t ask them…well apart from my friend whose daughter is a doctor so that sort of helps.

pp2

Third, family and friends played an important role in emotionally supporting patients by, for example, being an outlet to talk through acute episodes of LTCs or take their mind of the situation.

Okay they haven’t got a title as such, but yeah without yeah, without partner and children yeah I don’t know if I would have actually got through the mental rather than the physical sort of thing.

pp7

Finally, immediate family and partners were frequently mentioned to provide informal (social) care. Informal actors often were the ones mentioned under the category “other” SOCC.

Euhm, feeds me, I think the other aspect is that euh general hygiene of euh washing, ironing clothes and things like that…and euh, I mean general, generally helps me and I imagine she helps me more than I help her.

pp6

#### Experts: Condition-Specific Needs

The type(s) of experts involved in a PCN was dictated by the type of LTCs patients were diagnosed with. The role patients perceived experts to have, however, largely remained the same regardless of their specialism. According to the interviewees, specialists at the hospital were a source of disease-specific testing or monitoring and information.

I have to go and have my heart check and see that I’m alright. And I spent a lot of time in the hospital I know my way around there as well. You know (laughs) because I have to go to the heart clinic, the chest clinic, the blood place and then anything else. I mean I am forever...X-Ray, I mean you know so yes I know the hospital quite well.

pp1

#### Third Sector, Private Care, and Organizations

The fifth and final group that arose from the data was care provided by organizations, patient groups, etc. Third sector and charity organizations generally comprised services that were used as “substitutes” to health service care or ways to support needs that were not addressed elsewhere. As such, this group reflected a personalized addition to the PCN of patients with multimorbidity in accordance to their needs. Services included gardening and companies specialized in transportation for disabled patients.

Apart from substituting health service care, private care was also sought by patients that wanted timely advice or care.

And sometimes I, the person that I’ve seen, there’s a private physio, he’s, if I want it done quickly.

pp2

## Discussion

### Principal Findings

Our results draw a picture of a spread out and at times fragmented care system particularly challenging individuals with multimorbidity because of the need to facilitate information exchange among multiple stakeholders. Communication with, and between, providers constitutes a central challenge in care navigation. Most importantly, our results show that patients rely on a broad network spanning informal and formal care providers and also on public and private stakeholders, introducing barriers that extend beyond information sharing.

The major factor leading to a satisfying navigation experience that resided within the control of patients was an assertive, determined, and proactive approach. The system, for example, did not always allow patients to see the same provider. The way the care system was set up required participants to persevere in their navigation and deal with a number of complex barriers to accessing services, which could add frustration. Many times, patients felt they were sent “backward and forward” across the system. The finding that the quality of care was essentially dependent on the determination and ability of individual patients may lead to inequitable care. Those with less determination and poorer organizational skills appear to receive worse care. Thus, technology solutions must aim to fulfill these coordination functions, to ensure care is equitable for those who need it, not just those who ask loudest. Likewise, despite the significant role that informal caregivers play in the lives of patients with multimorbidity (eg, to facilitate attendance of appointments and support day-to-day care), many patients were aware of the burden they placed on these individuals involved in their care and were thus hesitant to place repeated demands on single individuals. While technology cannot solve some of the practical challenges of managing multiple LTCs, it can support patients in the management of their IC network, and possibly contribute to the reduction of informal caregiver burden by exploring how to effectively involve the wider community in care.

According to patients, the different parts of the care system formed separate entities. Smooth communication and interaction among different parts of the care system led to more satisfying navigation experiences. However, for many interviewees, it remained unclear whether this actually took place. Participants relied on their assumptions, as well as their experience, to judge this. On the level of provider-provider communication between colleagues, referral was mentioned as an indication that providers were interacting (eg, receiving copies of letters sent between providers) and was highly valued by patients. However, referrals do not include the extensive transfer of information; therefore, with an apparently limited crossover of information among professionals, it was frequently the patients’ task to bridge and connect the different parts of the system. Interestingly, existing technology largely focuses on the management of single diseases; for older adults with multiple LTCs to benefit from technology that supports care navigation (eg, mobile apps or Web-based logging solutions), an integrated approach that considers the complexity of the situation of an individual, how they manage their conditions, and seek to involve other stakeholders is required.

### Limitations and Future Work

There are some limitations and opportunities for future work that arise from this study. Most importantly, the outcomes of our mixed-methods approach to requirements analysis needs to be further validated by putting our findings into action: designing and implementing a care navigation tool to support older adults with multimorbidity. Furthermore, future work needs to consider the nature of our findings; questionnaire data were obtained from participants residing in England, and follow-up interviews were carried out with geographical restrictions, suggesting that findings need to be interpreted in this light and need to be reproduced on a national or international level to account for differences between care systems.

### Conclusions

This study stands at the intersection of care and technology, understanding the experience of care navigation for older adults with multimorbidity, as a step toward building technology to facilitate this process. We demonstrate that a mixed-methods approach can deliver insights across the breadth and depth of the care navigation process and outline complexities that need to be considered by both researchers and designers. Moving beyond care navigation, the detailed level of insight provided by the SNA and framework analysis highlights one of the core challenges for the human-computer interaction research in health care settings; while people see potential in the application of technology to care, they first and foremost want *better* care.

## Appendix

Multimedia Appendix 1 Questionnaire.

Multimedia Appendix 2 Interview schedule.

## Abbreviations

GP: general practitioner
HCC: health care actors in the community
HCH: health care actors at the hospital
IC: informal care
LTC: long-term condition
PCN: personal care network
SNA: social network analysis
SOCC: social care actors in the community
