# Supplementary material for: Understanding Care Navigation by Older Adults With Multimorbidity: Mixed-Methods Study Using Social Network and Framework Analyses
Source: JMIR Aging. 2018 Nov 14;1(2):e11054. doi: 10.2196/11054 (PMC6716432; doi:10.2196/11054)
Supplement: Multimedia Appendix 1 [file aging_v1i2e11054_app1.pdf]

UNIVERSITY OF LINCOLN

CaHRU
Community and Health Research Unit

Care Navigation Questionnaire

Research Team

Researcher	Affiliation
Jolien Vos	University of Lincoln
Dr Karen Windle	University of Lincoln
Prof Niroshan Siriwardena	University of Lincoln
Dr Conor Linehan	University College Cork
Dr Kathrin Gerling	University of Lincoln

Introduction

Dear participant,

Thank you for considering taking part in this study exploring ways to help older adults with multiple long term health conditions navigate the health and social care system. In this study we want to build a picture of your care network. We would like to hear your views about the different people involved in your care that are important to you or support you.

We are looking for participants who are:

- Aged 55 years or older
- Have at least two long term health conditions (e.g. diabetes and chronic lung disease)
- Living in England

We very much appreciate your support in this process. Attached you can find more details about the study.

If you have any questions, please contact Jolien Vos, researcher or Dr Karen Windle, project supervisor. Both are located in the School of Health and Social Care, Bridge House, University of Lincoln, Brayford Campus, Lincoln, LN6 7TS.
E-mail: jvos@lincoln.ac.uk/kwindle@lincoln.ac.uk. Telephone*:* 01522 886934

Once again, thank you for your consideration of taking part in this study.

Yours Sincerely,

Jolien

How to complete the questionnaire

You will be asked to respond to some questions about yourself and the people in your care network. There are no right or wrong answers and all the information will be anonymised and confidential.

Please answer the questions by:

- Ticking the box, like this ✓
- Or writing in the text box, like this [I enjoy gardening]

Completing the questionnaire is expected to take no longer than 20 minutes. Although the questionnaire involves several pages, it is likely that you can skip a lot of the sub questions.

After completion you can use the prepaid envelope in this study pack to send the questionnaire back to us.

About yourself

In this section you will be asked to answer questions that help us to gain an insight into which people are important to you in supporting you to live life as well as you can. There are no right or wrong answers.

1. a) What is your date of birth (DD/MM/YYYY):

_ _ / _ _ / _ _ _ _

b) What is your sex?

☐ Male
☐ Female
☐ Prefer not to say
☐ Other: ____

Please take a moment to think about all the different people you interact with *(friends /family / formal and informal carers)*

2. Which people are **important** to you for your **'social'** care? (Multiple answers are possible and allowed, please select all those who are relevant to your social care. Health care providers are discussed in the next section.)

☐ Community occupational therapist
☐ Family support worker
☐ Home care worker
☐ Social work assistant
☐ Social worker
☐ None of the above
☐ I don't know
☐ Other or social organisations (please specify)

3. For each of the below, please indicate the frequency, type and reason for contact with these people as well as the type of support they give you and how well this answers your needs.

a) Community occupational therapist

☐ I don't have any contact with this professional → Go to question 3.b)

Frequency of contact (choose 1)

☐ Every day
☐ Once or more a week, but not every day
☐ More than once a month, but not every week
☐ Once a month
☐ Every 1 or 2 months
☐ Every 3 or 4 months
☐ Every 5 or 6 months
☐ Every 7 to 12 months
☐ I don't know

Main way of contact (choose 1)

☐ Electronically (e.g., email)
☐ By telephone
☐ In person (face to face)

Reason for contact (choose 1)

☐ Short term problem not related to my chronic condition(s)
☐ Short term problem related to my chronic condition(s)
☐ Chronic condition

Main type of support provided (choose 1)

☐ Information and advice
☐ Emotional support
☐ Practical support
☐ Care (e.g., assessment, treatment, prescription)

Other types of support provided

☐ Information and advice
☐ Emotional support
☐ Practical support
☐ Care (e.g., assessment, treatment, prescription)

Level of satisfaction with support

☐ Very unsatisfied
☐ Unsatisfied
☐ Satisfied
☐ Very satisfied

Roughly how many different occupational therapists have you seen in the last 6 months?

b) Family support worker

☐ I don't have any contact with this professional → Go to question 3.c)

Frequency of contact (choose 1)

- ☐ Every day
- ☐ Once or more a week, but not every day
- ☐ More than once a month, but not every week
- ☐ Once a month
- ☐ Every 1 or 2 months
- ☐ Every 3 or 4 months
- ☐ Every 5 or 6 months
- ☐ Every 7 to 12 months
- ☐ I don't know

Main way of contact (choose 1)

- ☐ Electronically (e.g., email)
- ☐ By telephone
- ☐ In person (face to face)

Reason for contact (choose 1)

- ☐ Short term problem not related to my chronic condition(s)
- ☐ Short term problem related to my chronic condition(s)
- ☐ Chronic condition

Main type of support provided (choose 1)

- ☐ Information and advice
- ☐ Emotional support
- ☐ Practical support
- ☐ Care (e.g., assessment, treatment, prescription)

Other types of support provided

- ☐ Information and advice
- ☐ Emotional support
- ☐ Practical support
- ☐ Care (e.g., assessment, treatment, prescription)

Level of satisfaction with support

- ☐ Very unsatisfied
- ☐ Unsatisfied
- ☐ Satisfied
- ☐ Very satisfied

Roughly how many different family support workers have you seen in the last 6 months?

c) Home care worker

☐ I don't have any contact with this professional → Go to question 3.d)

Frequency of contact (choose 1)

- ☐ Every day
- ☐ Once or more a week, but not every day
- ☐ More than once a month, but not every week
- ☐ Once a month
- ☐ Every 1 or 2 months
- ☐ Every 3 or 4 months
- ☐ Every 5 or 6 months
- ☐ Every 7 to 12 months
- ☐ I don't know

Main way of contact (choose 1)

- ☐ Electronically (e.g., email)
- ☐ By telephone
- ☐ In person (face to face)

Reason for contact (choose 1)

- ☐ Short term problem not related to my chronic condition(s)
- ☐ Short term problem related to my chronic condition(s)
- ☐ Chronic condition

Main type of support provided (choose 1)

- ☐ Information and advice
- ☐ Emotional support
- ☐ Practical support
- ☐ Care (e.g., assessment, treatment, prescription)

Other types of support provided

- ☐ Information and advice
- ☐ Emotional support
- ☐ Practical support
- ☐ Care (e.g., assessment, treatment, prescription)

Level of satisfaction with support

- ☐ Very unsatisfied
- ☐ Unsatisfied
- ☐ Satisfied
- ☐ Very satisfied

Roughly how many different home care workers have you seen in the last 6 months?

d) Social work assistant

☐ I don't have any contact with this professional → Go to question 3.e)

Frequency of contact (choose 1)

☐ Every day
☐ Once or more a week, but not every day
☐ More than once a month, but not every week
☐ Once a month
☐ Every 1 or 2 months
☐ Every 3 or 4 months
☐ Every 5 or 6 months
☐ Every 7 to 12 months
☐ I don't know

Main way of contact (choose 1)

☐ Electronically (e.g., email)
☐ By telephone
☐ In person (face to face)

Reason for contact (choose 1)

☐ Short term problem not related to my chronic condition(s)
☐ Short term problem related to my chronic condition(s)
☐ Chronic condition

Main type of support provided (choose 1)

☐ Information and advice
☐ Emotional support
☐ Practical support
☐ Care (e.g., assessment, treatment, prescription)

Other types of support provided

☐ Information and advice
☐ Emotional support
☐ Practical support
☐ Care (e.g., assessment, treatment, prescription)

Level of satisfaction with support

☐ Very unsatisfied
☐ Unsatisfied
☐ Satisfied
☐ Very satisfied

Roughly how many different social work assistants have you seen in the last 6 months?

______

e) Social worker

☐ I don't have any contact with this professional → Go to question 3.f)

Frequency of contact (choose 1)

☐ Every day
☐ Once or more a week, but not every day
☐ More than once a month, but not every week
☐ Once a month
☐ Every 1 or 2 months
☐ Every 3 or 4 months
☐ Every 5 or 6 months
☐ Every 7 to 12 months
☐ I don't know

Main way of contact (choose 1)

☐ Electronically (e.g., email)
☐ By telephone
☐ In person (face to face)

Reason for contact (choose 1)

☐ Short term problem not related to my chronic condition(s)
☐ Short term problem related to my chronic condition(s)
☐ Chronic condition

Main type of support provided (choose 1)

☐ Information and advice
☐ Emotional support
☐ Practical support
☐ Care (e.g., assessment, treatment, prescription)

Other types of support provided

☐ Information and advice
☐ Emotional support
☐ Practical support
☐ Care (e.g., assessment, treatment, prescription)

Level of satisfaction with support

☐ Very unsatisfied
☐ Unsatisfied
☐ Satisfied
☐ Very satisfied

Roughly how many different social workers have you seen in the last 6 months?

f) Other or organisations (please specify:_______________________________)

☐ N/A → Go to the next question

Frequency of contact (choose 1)

☐ Every day
☐ Once or more a week, but not every day
☐ More than once a month, but not every week
☐ Once a month
☐ Every 1 or 2 months
☐ Every 3 or 4 months
☐ Every 5 or 6 months
☐ Every 7 to 12 months
☐ I don't know

Main way of contact (choose 1)

☐ Electronically (e.g., email)
☐ By telephone
☐ In person (face to face)

Reason for contact (choose 1)

☐ Short term problem not related to my chronic condition(s)
☐ Short term problem related to my chronic condition(s)
☐ Chronic condition

Main type of support provided (choose 1)

☐ Information and advice
☐ Emotional support
☐ Practical support
☐ Care (e.g., assessment, treatment, prescription)

Other types of support provided

☐ Information and advice
☐ Emotional support
☐ Practical support
☐ Care (e.g., assessment, treatment, prescription)

Level of satisfaction with support

☐ Very unsatisfied
☐ Unsatisfied
☐ Satisfied
☐ Very satisfied

Roughly how many different "others" have you seen in the last 6 months?

4. On average, how many different professionals did you see with regard to your ‘social’ care in the last 6 months?

- ☐ 1
- ☐ 2
- ☐ 3
- ☐ 4
- ☐ 5
- ☐ 6
- ☐ 7
- ☐ 8
- ☐ 9
- ☐ 10
- ☐ More than

5. Which people, based in the community (i.e. outside the hospital), are **important** to you for your ‘**health**’ care? (Multiple answers are possible and allowed, please select all those who are relevant to you. Professionals based in the hospital are included in the next section)

- ☐ Clinical psychologist
- ☐ Clinical support worker nursing
- ☐ Community chiropodist/podiatrist
- ☐ Community nurse (district nurse)/ nurse specialist
- ☐ (Community) pharmacist
- ☐ Community physiotherapist
- ☐ Community speech and language therapist
- ☐ Dentist
- ☐ General practitioner (GP)
- ☐ Health visitor
- ☐ NHS community occupational therapist
- ☐ Nurse (GP practice)
- ☐ Nurse (mental health)
- ☐ None of the above
- ☐ I don’t know
- ☐ Others or organisations (please specify)

6. For each of the below, please indicate the frequency, type and reason for contact with these people as well as the type of support they give you and how well this answers your needs.

a) Clinical psychologist

☐ I don't have any contact with this professional → Go to question 6.b)

Frequency of contact (choose 1)

☐ Every day
☐ Once or more a week, but not every day
☐ More than once a month, but not every week
☐ Once a month
☐ Every 1 or 2 months
☐ Every 3 or 4 months
☐ Every 5 or 6 months
☐ Every 7 to 12 months
☐ I don't know

Main way of contact (choose 1)

☐ Electronically (e.g., email)
☐ By telephone
☐ In person (face to face)

Reason for contact (choose 1)

☐ Short term problem not related to my chronic condition(s)
☐ Short term problem related to my chronic condition(s)
☐ Chronic condition

Main type of support provided (choose 1)

☐ Information and advice
☐ Emotional support
☐ Practical support
☐ Care (e.g., assessment, treatment, prescription)

Other types of support provided

☐ Information and advice
☐ Emotional support
☐ Practical support
☐ Care (e.g., assessment, treatment, prescription)

Level of satisfaction with support

☐ Very unsatisfied
☐ Unsatisfied
☐ Satisfied
☐ Very satisfied

Roughly how many different clinical psychologists have you seen in the last 6 months?

____

b) Clinical support worker nursing

☐ I don't have any contact with this professional → Go to question 6.c)

Frequency of contact (choose 1)

- ☐ Every day
- ☐ Once or more a week, but not every day
- ☐ More than once a month, but not every week
- ☐ Once a month
- ☐ Every 1 or 2 months
- ☐ Every 3 or 4 months
- ☐ Every 5 or 6 months
- ☐ Every 7 to 12 months
- ☐ I don't know

Main way of contact (choose 1)

- ☐ Electronically (e.g., email)
- ☐ By telephone
- ☐ In person (face to face)

Reason for contact (choose 1)

- ☐ Short term problem not related to my chronic condition(s)
- ☐ Short term problem related to my chronic condition(s)
- ☐ Chronic condition

Main type of support provided (choose 1)

- ☐ Information and advice
- ☐ Emotional support
- ☐ Practical support
- ☐ Care (e.g., assessment, treatment, prescription)

Other types of support provided

- ☐ Information and advice
- ☐ Emotional support
- ☐ Practical support
- ☐ Care (e.g., assessment, treatment, prescription)

Level of satisfaction with support

- ☐ Very unsatisfied
- ☐ Unsatisfied
- ☐ Satisfied
- ☐ Very satisfied

Roughly how many different clinical support workers have you seen in the last 6 months?

c) Community chiropodist/podiatrist

☐ I don't have any contact with this professional → Go to question 6.d)

Frequency of contact (choose 1)

- ☐ Every day
- ☐ Once or more a week, but not every day
- ☐ More than once a month, but not every week
- ☐ Once a month
- ☐ Every 1 or 2 months
- ☐ Every 3 or 4 months
- ☐ Every 5 or 6 months
- ☐ Every 7 to 12 months
- ☐ I don't know

Main way of contact (choose 1)

- ☐ Electronically (e.g., email)
- ☐ By telephone
- ☐ In person (face to face)

Reason for contact (choose 1)

- ☐ Short term problem not related to my chronic condition(s)
- ☐ Short term problem related to my chronic condition(s)
- ☐ Chronic condition

Main type of support provided (choose 1)

- ☐ Information and advice
- ☐ Emotional support
- ☐ Practical support
- ☐ Care (e.g., assessment, treatment, prescription)

Other types of support provided

- ☐ Information and advice
- ☐ Emotional support
- ☐ Practical support
- ☐ Care (e.g., assessment, treatment, prescription)

Level of satisfaction with support

- ☐ Very unsatisfied
- ☐ Unsatisfied
- ☐ Satisfied
- ☐ Very satisfied

Roughly how many different chiropodists/podiatrists have you seen in the last 6 months?

d) Community nurse (district nurse)/ specialist nurse

☐ I don't have any contact with this professional → Go to question 6.e)

Frequency of contact (choose 1)

- ☐ Every day
- ☐ Once or more a week, but not every day
- ☐ More than once a month, but not every week
- ☐ Once a month
- ☐ Every 1 or 2 months
- ☐ Every 3 or 4 months
- ☐ Every 5 or 6 months
- ☐ Every 7 to 12 months
- ☐ I don't know

Main way of contact (choose 1)

- ☐ Electronically (e.g., email)
- ☐ By telephone
- ☐ In person (face to face)

Reason for contact (choose 1)

- ☐ Short term problem not related to my chronic condition(s)
- ☐ Short term problem related to my chronic condition(s)
- ☐ Chronic condition

Main type of support provided (choose 1)

- ☐ Information and advice
- ☐ Emotional support
- ☐ Practical support
- ☐ Care (e.g., assessment, treatment, prescription)

Other types of support provided

- ☐ Information and advice
- ☐ Emotional support
- ☐ Practical support
- ☐ Care (e.g., assessment, treatment, prescription)

Level of satisfaction with support

- ☐ Very unsatisfied
- ☐ Unsatisfied
- ☐ Satisfied
- ☐ Very satisfied

Roughly how many different community nurses have you seen in the last 6 months?

e) (Community) pharmacist

☐ I don't have any contact with this professional → Go to question 6.f)

Frequency of contact

- ☐ Every day
- ☐ Once or more a week, but not every day
- ☐ More than once a month, but not every week
- ☐ Once a month
- ☐ Every 1 or 2 months
- ☐ Every 3 or 4 months
- ☐ Every 5 or 6 months
- ☐ Every 7 to 12 months
- ☐ I don't know

Main way of contact

- ☐ Electronically (e.g., email)
- ☐ By telephone
- ☐ In person (face to face)

Reason for contact

- ☐ Short term problem not related to my chronic condition(s)
- ☐ Short term problem related to my chronic condition(s)
- ☐ Chronic condition

Main type of support provided

- ☐ Information and advice
- ☐ Emotional support
- ☐ Practical support
- ☐ Care (e.g., assessment, treatment, prescription)

Other types of support provided

- ☐ Information and advice
- ☐ Emotional support
- ☐ Practical support
- ☐ Care (e.g., assessment, treatment, prescription)

Level of satisfaction with support

- ☐ Very unsatisfied
- ☐ Unsatisfied
- ☐ Satisfied
- ☐ Very satisfied

Roughly how many different (community) pharmacists have you seen in the last 6 months?

f) Community physiotherapist

☐ I don't have any contact with this professional → Go to question 6.g)

Frequency of contact (choose 1)

- ☐ Every day
- ☐ Once or more a week, but not every day
- ☐ More than once a month, but not every week
- ☐ Once a month
- ☐ Every 1 or 2 months
- ☐ Every 3 or 4 months
- ☐ Every 5 or 6 months
- ☐ Every 7 to 12 months
- ☐ I don't know

Main way of contact (choose 1)

- ☐ Electronically (e.g., email)
- ☐ By telephone
- ☐ In person (face to face)

Reason for contact (choose 1)

- ☐ Short term problem not related to my chronic condition(s)
- ☐ Short term problem related to my chronic condition(s)
- ☐ Chronic condition

Main type of support provided (choose 1)

- ☐ Information and advice
- ☐ Emotional support
- ☐ Practical support
- ☐ Care (e.g., assessment, treatment, prescription)

Other types of support provided

- ☐ Information and advice
- ☐ Emotional support
- ☐ Practical support
- ☐ Care (e.g., assessment, treatment, prescription)

Level of satisfaction with support

- ☐ Very unsatisfied
- ☐ Unsatisfied
- ☐ Satisfied
- ☐ Very satisfied

Roughly how many different physiotherapists have you seen in the last 6 months?

[ ]

g) Community speech and language therapist

☐ I don't have any contact with this professional → Go to question 6.h)

Frequency of contact (choose 1)

- ☐ Every day
- ☐ Once or more a week, but not every day
- ☐ More than once a month, but not every week
- ☐ Once a month
- ☐ Every 1 or 2 months
- ☐ Every 3 or 4 months
- ☐ Every 5 or 6 months
- ☐ Every 7 to 12 months
- ☐ I don't know

Main way of contact (choose 1)

- ☐ Electronically (e.g., email)
- ☐ By telephone
- ☐ In person (face to face)

Reason for contact (choose 1)

- ☐ Short term problem not related to my chronic condition(s)
- ☐ Short term problem related to my chronic condition(s)
- ☐ Chronic condition

Main type of support provided (choose 1)

- ☐ Information and advice
- ☐ Emotional support
- ☐ Practical support
- ☐ Care (e.g., assessment, treatment, prescription)

Other types of support provided

- ☐ Information and advice
- ☐ Emotional support
- ☐ Practical support
- ☐ Care (e.g., assessment, treatment, prescription)

Level of satisfaction with support

- ☐ Very unsatisfied
- ☐ Unsatisfied
- ☐ Satisfied
- ☐ Very satisfied

Roughly how many different speech and language therapists have you seen in the last 6 months?

[ ]

h) Dentist

☐ I don't have any contact with this professional → Go to question 6.i)

Frequency of contact (choose 1)

☐ Every day
☐ Once or more a week, but not every day
☐ More than once a month, but not every week
☐ Once a month
☐ Every 1 or 2 months
☐ Every 3 or 4 months
☐ Every 5 or 6 months
☐ Every 7 to 12 months
☐ I don't know

Main way of contact (choose 1)

☐ Electronically (e.g., email)
☐ By telephone
☐ In person (face to face)

Reason for contact (choose 1)

☐ Short term problem not related to my chronic condition(s)
☐ Short term problem related to my chronic condition(s)
☐ Chronic condition

Main type of support provided (choose 1)

☐ Information and advice
☐ Emotional support
☐ Practical support
☐ Care (e.g., assessment, treatment, prescription)

Other types of support provided

☐ Information and advice
☐ Emotional support
☐ Practical support
☐ Care (e.g., assessment, treatment, prescription)

Level of satisfaction with support

☐ Very unsatisfied
☐ Unsatisfied
☐ Satisfied
☐ Very satisfied

Roughly how many different dentists have you seen in the last 6 months?

i) General practitioner (GP)

☐ I don't have any contact with this professional → Go to question 6.j)

Frequency of contact (choose 1)

- ☐ Every day
- ☐ Once or more a week, but not every day
- ☐ More than once a month, but not every week
- ☐ Once a month
- ☐ Every 1 or 2 months
- ☐ Every 3 or 4 months
- ☐ Every 5 or 6 months
- ☐ Every 7 to 12 months
- ☐ I don't know

Main way of contact (choose 1)

- ☐ Electronically (e.g., email)
- ☐ By telephone
- ☐ In person (face to face)

Reason for contact (choose 1)

- ☐ Short term problem not related to my chronic condition(s)
- ☐ Short term problem related to my chronic condition(s)
- ☐ Chronic condition

Main type of support provided (choose 1)

- ☐ Information and advice
- ☐ Emotional support
- ☐ Practical support
- ☐ Care (e.g., assessment, treatment, prescription)

Other types of support provided

- ☐ Information and advice
- ☐ Emotional support
- ☐ Practical support
- ☐ Care (e.g., assessment, treatment, prescription)

Level of satisfaction with support

- ☐ Very unsatisfied
- ☐ Unsatisfied
- ☐ Satisfied
- ☐ Very satisfied

Roughly how many different GPs have you seen in the last 6 months?

j) Health visitor

☐ I don't have any contact with this professional → Go to question 6.k)

Frequency of contact (choose 1)

☐ Every day
☐ Once or more a week, but not every day
☐ More than once a month, but not every week
☐ Once a month
☐ Every 1 or 2 months
☐ Every 3 or 4 months
☐ Every 5 or 6 months
☐ Every 7 to 12 months
☐ I don't know

Main way of contact (choose 1)

☐ Electronically (e.g., email)
☐ By telephone
☐ In person (face to face)

Reason for contact (choose 1)

☐ Short term problem not related to my chronic condition(s)
☐ Short term problem related to my chronic condition(s)
☐ Chronic condition

Main type of support provided (choose 1)

☐ Information and advice
☐ Emotional support
☐ Practical support
☐ Care (e.g., assessment, treatment, prescription)

Other types of support provided

☐ Information and advice
☐ Emotional support
☐ Practical support
☐ Care (e.g., assessment, treatment, prescription)

Level of satisfaction with support

☐ Very unsatisfied
☐ Unsatisfied
☐ Satisfied
☐ Very satisfied

Roughly how many different health visitors have you seen in the last 6 months?

k) Community occupational therapist

☐ I don't have any contact with this professional → Go to question 6.l)

Frequency of contact (choose 1)

- ☐ Every day
- ☐ Once or more a week, but not every day
- ☐ More than once a month, but not every week
- ☐ Once a month
- ☐ Every 1 or 2 months
- ☐ Every 3 or 4 months
- ☐ Every 5 or 6 months
- ☐ Every 7 to 12 months
- ☐ I don't know

Main way of contact (choose 1)

- ☐ Electronically (e.g., email)
- ☐ By telephone
- ☐ In person (face to face)

Reason for contact (choose 1)

- ☐ Short term problem not related to my chronic condition(s)
- ☐ Short term problem related to my chronic condition(s)
- ☐ Chronic condition

Main type of support provided (choose 1)

- ☐ Information and advice
- ☐ Emotional support
- ☐ Practical support
- ☐ Care (e.g., assessment, treatment, prescription)

Other types of support provided

- ☐ Information and advice
- ☐ Emotional support
- ☐ Practical support
- ☐ Care (e.g., assessment, treatment, prescription)

Level of satisfaction with support

- ☐ Very unsatisfied
- ☐ Unsatisfied
- ☐ Satisfied
- ☐ Very satisfied

Roughly how many different occupational therapists have you seen in the last 6 months?

[ ]

l) Nurse (GP practice)

☐ I don't have any contact with this professional → Go to question 6.m)

Frequency of contact (choose 1)

☐ Every day
☐ Once or more a week, but not every day
☐ More than once a month, but not every week
☐ Once a month
☐ Every 1 or 2 months
☐ Every 3 or 4 months
☐ Every 5 or 6 months
☐ Every 7 to 12 months
☐ I don't know

Main way of contact (choose 1)

☐ Electronically (e.g., email)
☐ By telephone
☐ In person (face to face)

Reason for contact (choose 1)

☐ Short term problem not related to my chronic condition(s)
☐ Short term problem related to my chronic condition(s)
☐ Chronic condition

Main type of support provided (choose 1)

☐ Information and advice
☐ Emotional support
☐ Practical support
☐ Care (e.g., assessment, treatment, prescription)

Other types of support provided

☐ Information and advice
☐ Emotional support
☐ Practical support
☐ Care (e.g., assessment, treatment, prescription)

Level of satisfaction with support

☐ Very unsatisfied
☐ Unsatisfied
☐ Satisfied
☐ Very satisfied

Roughly how many different GP nurses have you seen in the last 6 months?

m) Nurse (mental health)

☐ I don't have any contact with this professional → Go to question 6.n)

Frequency of contact (choose 1)

☐ Every day
☐ Once or more a week, but not every day
☐ More than once a month, but not every week
☐ Once a month
☐ Every 1 or 2 months
☐ Every 3 or 4 months
☐ Every 5 or 6 months
☐ Every 7 to 12 months
☐ I don't know

Main way of contact (choose 1)

☐ Electronically (e.g., email)
☐ By telephone
☐ In person (face to face)

Reason for contact (choose 1)

☐ Short term problem not related to my chronic condition(s)
☐ Short term problem related to my chronic condition(s)
☐ Chronic condition

Main type of support provided (choose 1)

☐ Information and advice
☐ Emotional support
☐ Practical support
☐ Care (e.g., assessment, treatment, prescription)

Other types of support provided

☐ Information and advice
☐ Emotional support
☐ Practical support
☐ Care (e.g., assessment, treatment, prescription)

Level of satisfaction with support

☐ Very unsatisfied
☐ Unsatisfied
☐ Satisfied
☐ Very satisfied

Roughly how many different mental health nurses have you seen in the last 6 months?

n) Others or organisations (please specify:______________________________)

☐ I don't have any contact with this professional → Go to the next question

Frequency of contact (choose 1)

☐ Every day
☐ Once or more a week, but not every day
☐ More than once a month, but not every week
☐ Once a month
☐ Every 1 or 2 months
☐ Every 3 or 4 months
☐ Every 5 or 6 months
☐ Every 7 to 12 months
☐ I don't know

Main way of contact (choose 1)

☐ Electronically (e.g., email)
☐ By telephone
☐ In person (face to face)

Reason for contact (choose 1)

☐ Short term problem not related to my chronic condition(s)
☐ Short term problem related to my chronic condition(s)
☐ Chronic condition

Main type of support provided (choose 1)

☐ Information and advice
☐ Emotional support
☐ Practical support
☐ Care (e.g., assessment, treatment, prescription)

Other types of support provided

☐ Information and advice
☐ Emotional support
☐ Practical support
☐ Care (e.g., assessment, treatment, prescription)

Level of satisfaction with support

☐ Very unsatisfied
☐ Unsatisfied
☐ Satisfied
☐ Very satisfied

Roughly how many different "others" have you seen in the last 6 months?

7. On average, how many different professionals based in the community did you see with regard to your ‘health’ care in the last 6 months?

- ☐ 1
- ☐ 2
- ☐ 3
- ☐ 4
- ☐ 5
- ☐ 6
- ☐ 7
- ☐ 8
- ☐ 9
- ☐ 10
- ☐ More than 10

8. Which people, <u>based in the hospital</u>, are **important** to you for your **‘health’** care? (Multiple answers are possible and allowed, please select all those who are relevant to you.)

- ☐ Allied health professional support worker
- ☐ (Associate) specialist
- ☐ Clinical support worker
- ☐ Hospital dietitian
- ☐ Hospital nurse
- ☐ Hospital occupational therapist
- ☐ Hospital pharmacist
- ☐ Hospital physiotherapist
- ☐ Hospital radiographer
- ☐ Hospital speech and language therapist
- ☐ Medical consultant
- ☐ Psychiatric consultant
- ☐ Surgical consultant
- ☐ Other hospital doctors
- ☐ None of the above
- ☐ I don’t know
- ☐ Others or organisations (please specify)

9. For each of the below, please indicate the frequency, type and reason for contact with these people as well as the type of support they give you and how well this answers your needs.

a) Allied health professional support worker

☐ I don't have any contact with this professional → Go to question 9.b)

Frequency of contact (choose 1)

- ☐ Every day
- ☐ Once or more a week, but not every day
- ☐ More than once a month, but not every week
- ☐ Once a month
- ☐ Every 1 or 2 months
- ☐ Every 3 or 4 months
- ☐ Every 5 or 6 months
- ☐ Every 7 to 12 months
- ☐ I don't know

Main way of contact (choose 1)

- ☐ Electronically (e.g., email)
- ☐ By telephone
- ☐ In person (face to face)

Reason for contact (choose 1)

- ☐ Short term problem not related to my chronic condition(s)
- ☐ Short term problem related to my chronic condition(s)
- ☐ Chronic condition

Main type of support provided (choose 1)

- ☐ Information and advice
- ☐ Emotional support
- ☐ Practical support
- ☐ Care (e.g., assessment, treatment, prescription)

Other types of support provided

- ☐ Information and advice
- ☐ Emotional support
- ☐ Practical support
- ☐ Care (e.g., assessment, treatment, prescription)

Level of satisfaction with support

- ☐ Very unsatisfied
- ☐ Unsatisfied
- ☐ Satisfied
- ☐ Very satisfied

Roughly how many different allied health professional support workers have you seen in the last 6 months?

b) (Associate) specialist

☐ I don't have any contact with this professional → Go to question 9.c)

Frequency of contact (choose 1)

- ☐ Every day
- ☐ Once or more a week, but not every day
- ☐ More than once a month, but not every week
- ☐ Once a month
- ☐ Every 1 or 2 months
- ☐ Every 3 or 4 months
- ☐ Every 5 or 6 months
- ☐ Every 7 to 12 months
- ☐ I don't know

Main way of contact (choose 1)

- ☐ Electronically (e.g., email)
- ☐ By telephone
- ☐ In person (face to face)

Reason for contact (choose 1)

- ☐ Short term problem not related to my chronic condition(s)
- ☐ Short term problem related to my chronic condition(s)
- ☐ Chronic condition

Main type of support provided (choose 1)

- ☐ Information and advice
- ☐ Emotional support
- ☐ Practical support
- ☐ Care (e.g., assessment, treatment, prescription)

Other types of support provided

- ☐ Information and advice
- ☐ Emotional support
- ☐ Practical support
- ☐ Care (e.g., assessment, treatment, prescription)

Level of satisfaction with support

- ☐ Very unsatisfied
- ☐ Unsatisfied
- ☐ Satisfied
- ☐ Very satisfied

Roughly how many different (associate) specialists have you seen in the last 6 months?

c) Clinical support worker

☐ I don't have any contact with this professional → Go to question 9.d)

Frequency of contact (choose 1)

- ☐ Every day
- ☐ Once or more a week, but not every day
- ☐ More than once a month, but not every week
- ☐ Once a month
- ☐ Every 1 or 2 months
- ☐ Every 3 or 4 months
- ☐ Every 5 or 6 months
- ☐ Every 7 to 12 months
- ☐ I don't know

Main way of contact (choose 1)

- ☐ Electronically (e.g., email)
- ☐ By telephone
- ☐ In person (face to face)

Reason for contact (choose 1)

- ☐ Short term problem not related to my chronic condition(s)
- ☐ Short term problem related to my chronic condition(s)
- ☐ Chronic condition

Main type of support provided (choose 1)

- ☐ Information and advice
- ☐ Emotional support
- ☐ Practical support
- ☐ Care (e.g., assessment, treatment, prescription)

Other types of support provided

- ☐ Information and advice
- ☐ Emotional support
- ☐ Practical support
- ☐ Care (e.g., assessment, treatment, prescription)

Level of satisfaction with support

- ☐ Very unsatisfied
- ☐ Unsatisfied
- ☐ Satisfied
- ☐ Very satisfied

Roughly how many different clinical support workers have you seen in the last 6 months?

d) Hospital dietitian

☐ I don't have any contact with this professional → Go to question 9.e)

Frequency of contact (choose 1)

- ☐ Every day
- ☐ Once or more a week, but not every day
- ☐ More than once a month, but not every week
- ☐ Once a month
- ☐ Every 1 or 2 months
- ☐ Every 3 or 4 months
- ☐ Every 5 or 6 months
- ☐ Every 7 to 12 months
- ☐ I don't know

Main way of contact (choose 1)

- ☐ Electronically (e.g., email)
- ☐ By telephone
- ☐ In person (face to face)

Reason for contact (choose 1)

- ☐ Short term problem not related to my chronic condition(s)
- ☐ Short term problem related to my chronic condition(s)
- ☐ Chronic condition

Main type of support provided (choose 1)

- ☐ Information and advice
- ☐ Emotional support
- ☐ Practical support
- ☐ Care (e.g., assessment, treatment, prescription)

Other types of support provided

- ☐ Information and advice
- ☐ Emotional support
- ☐ Practical support
- ☐ Care (e.g., assessment, treatment, prescription)

Level of satisfaction with support

- ☐ Very unsatisfied
- ☐ Unsatisfied
- ☐ Satisfied
- ☐ Very satisfied

Roughly how many different hospital dietitians have you seen in the last 6 months?

e) Hospital nurse

☐ I don't have any contact with this professional → Go to question 9.f)

Frequency of contact (choose 1)

☐ Every day
☐ Once or more a week, but not every day
☐ More than once a month, but not every week
☐ Once a month
☐ Every 1 or 2 months
☐ Every 3 or 4 months
☐ Every 5 or 6 months
☐ Every 7 to 12 months
☐ I don't know

Main way of contact (choose 1)

☐ Electronically (e.g., email)
☐ By telephone
☐ In person (face to face)

Reason for contact (choose 1)

☐ Short term problem not related to my chronic condition(s)
☐ Short term problem related to my chronic condition(s)
☐ Chronic condition

Main type of support provided (choose 1)

☐ Information and advice
☐ Emotional support
☐ Practical support
☐ Care (e.g., assessment, treatment, prescription)

Other types of support provided

☐ Information and advice
☐ Emotional support
☐ Practical support
☐ Care (e.g., assessment, treatment, prescription)

Level of satisfaction with support

☐ Very unsatisfied
☐ Unsatisfied
☐ Satisfied
☐ Very satisfied

Roughly how many different hospital nurses have you seen in the last 6 months?

f) Hospital occupational therapist

☐ I don't have any contact with this professional → Go to question 9.g)

Frequency of contact (choose 1)

☐ Every day
☐ Once or more a week, but not every day
☐ More than once a month, but not every week
☐ Once a month
☐ Every 1 or 2 months
☐ Every 3 or 4 months
☐ Every 5 or 6 months
☐ Every 7 to 12 months
☐ I don't know

Main way of contact (choose 1)

☐ Electronically (e.g., email)
☐ By telephone
☐ In person (face to face)

Reason for contact (choose 1)

☐ Short term problem not related to my chronic condition(s)
☐ Short term problem related to my chronic condition(s)
☐ Chronic condition

Main type of support provided (choose 1)

☐ Information and advice
☐ Emotional support
☐ Practical support
☐ Care (e.g., assessment, treatment, prescription)

Other types of support provided

☐ Information and advice
☐ Emotional support
☐ Practical support
☐ Care (e.g., assessment, treatment, prescription)

Level of satisfaction with support

☐ Very unsatisfied
☐ Unsatisfied
☐ Satisfied
☐ Very satisfied

Roughly how many different occupational therapists have you seen in the last 6 months?

g) Hospital pharmacist

☐ I don't have any contact with this professional → Go to question 9.h)

Frequency of contact (choose 1)

- ☐ Every day
- ☐ Once or more a week, but not every day
- ☐ More than once a month, but not every week
- ☐ Once a month
- ☐ Every 1 or 2 months
- ☐ Every 3 or 4 months
- ☐ Every 5 or 6 months
- ☐ Every 7 to 12 months
- ☐ I don't know

Main way of contact (choose 1)

- ☐ Electronically (e.g., email)
- ☐ By telephone
- ☐ In person (face to face)

Reason for contact (choose 1)

- ☐ Short term problem not related to my chronic condition(s)
- ☐ Short term problem related to my chronic condition(s)
- ☐ Chronic condition

Main type of support provided (choose 1)

- ☐ Information and advice
- ☐ Emotional support
- ☐ Practical support
- ☐ Care (e.g., assessment, treatment, prescription)

Other types of support provided

- ☐ Information and advice
- ☐ Emotional support
- ☐ Practical support
- ☐ Care (e.g., assessment, treatment, prescription)

Level of satisfaction with support

- ☐ Very unsatisfied
- ☐ Unsatisfied
- ☐ Satisfied
- ☐ Very satisfied

Roughly how many different hospital pharmacists have you seen in the last 6 months?

h) Hospital physiotherapist

☐ I don't have any contact with this professional → Go to question 9.i)

Frequency of contact (choose 1)

- ☐ Every day
- ☐ Once or more a week, but not every day
- ☐ More than once a month, but not every week
- ☐ Once a month
- ☐ Every 1 or 2 months
- ☐ Every 3 or 4 months
- ☐ Every 5 or 6 months
- ☐ Every 7 to 12 months
- ☐ I don't know

Main way of contact (choose 1)

- ☐ Electronically (e.g., email)
- ☐ By telephone
- ☐ In person (face to face)

Reason for contact (choose 1)

- ☐ Short term problem not related to my chronic condition(s)
- ☐ Short term problem related to my chronic condition(s)
- ☐ Chronic condition

Main type of support provided (choose 1)

- ☐ Information and advice
- ☐ Emotional support
- ☐ Practical support
- ☐ Care (e.g., assessment, treatment, prescription)

Other types of support provided

- ☐ Information and advice
- ☐ Emotional support
- ☐ Practical support
- ☐ Care (e.g., assessment, treatment, prescription)

Level of satisfaction with support

- ☐ Very unsatisfied
- ☐ Unsatisfied
- ☐ Satisfied
- ☐ Very satisfied

Roughly how many different hospital physiotherapists have you seen in the last 6 months?

i) Hospital radiographer

☐ I don't have any contact with this professional → Go to question 9.j)

Frequency of contact (choose 1)

- ☐ Every day
- ☐ Once or more a week, but not every day
- ☐ More than once a month, but not every week
- ☐ Once a month
- ☐ Every 1 or 2 months
- ☐ Every 3 or 4 months
- ☐ Every 5 or 6 months
- ☐ Every 7 to 12 months
- ☐ I don't know

Main way of contact (choose 1)

- ☐ Electronically (e.g., email)
- ☐ By telephone
- ☐ In person (face to face)

Reason for contact (choose 1)

- ☐ Short term problem not related to my chronic condition(s)
- ☐ Short term problem related to my chronic condition(s)
- ☐ Chronic condition

Main type of support provided (choose 1)

- ☐ Information and advice
- ☐ Emotional support
- ☐ Practical support
- ☐ Care (e.g., assessment, treatment, prescription)

Other types of support provided

- ☐ Information and advice
- ☐ Emotional support
- ☐ Practical support
- ☐ Care (e.g., assessment, treatment, prescription)

Level of satisfaction with support

- ☐ Very unsatisfied
- ☐ Unsatisfied
- ☐ Satisfied
- ☐ Very satisfied

Roughly how many different hospital radiographers have you seen in the last 6 months?

j) Hospital speech and language therapist

☐ I don't have any contact with this professional → Go to question 9.k)

Frequency of contact (choose 1)

- ☐ Every day
- ☐ Once or more a week, but not every day
- ☐ More than once a month, but not every week
- ☐ Once a month
- ☐ Every 1 or 2 months
- ☐ Every 3 or 4 months
- ☐ Every 5 or 6 months
- ☐ Every 7 to 12 months
- ☐ I don't know

Main way of contact (choose 1)

- ☐ Electronically (e.g., email)
- ☐ By telephone
- ☐ In person (face to face)

Reason for contact (choose 1)

- ☐ Short term problem not related to my chronic condition(s)
- ☐ Short term problem related to my chronic condition(s)
- ☐ Chronic condition

Main type of support provided (choose 1)

- ☐ Information and advice
- ☐ Emotional support
- ☐ Practical support
- ☐ Care (e.g., assessment, treatment, prescription)

Other types of support provided

- ☐ Information and advice
- ☐ Emotional support
- ☐ Practical support
- ☐ Care (e.g., assessment, treatment, prescription)

Level of satisfaction with support

- ☐ Very unsatisfied
- ☐ Unsatisfied
- ☐ Satisfied
- ☐ Very satisfied

Roughly how many different speech and language therapists have you seen in the last 6 months?

k) Medical consultant

☐ I don't have any contact with this professional → Go to question 9.l)

Frequency of contact (choose 1)

☐ Every day
☐ Once or more a week, but not every day
☐ More than once a month, but not every week
☐ Once a month
☐ Every 1 or 2 months
☐ Every 3 or 4 months
☐ Every 5 or 6 months
☐ Every 7 to 12 months
☐ I don't know

Main way of contact (choose 1)

☐ Electronically (e.g., email)
☐ By telephone
☐ In person (face to face)

Reason for contact (choose 1)

☐ Short term problem not related to my chronic condition(s)
☐ Short term problem related to my chronic condition(s)
☐ Chronic condition

Main type of support provided (choose 1)

☐ Information and advice
☐ Emotional support
☐ Practical support
☐ Care (e.g., assessment, treatment, prescription)

Other types of support provided

☐ Information and advice
☐ Emotional support
☐ Practical support
☐ Care (e.g., assessment, treatment, prescription)

Level of satisfaction with support

☐ Very unsatisfied
☐ Unsatisfied
☐ Satisfied
☐ Very satisfied

Roughly how many different medical consultants have you seen in the last 6 months?

l) Psychiatric consultant

☐ I don't have any contact with this professional → Go to question 9.m)

Frequency of contact (choose 1)

- ☐ Every day
- ☐ Once or more a week, but not every day
- ☐ More than once a month, but not every week
- ☐ Once a month
- ☐ Every 1 or 2 months
- ☐ Every 3 or 4 months
- ☐ Every 5 or 6 months
- ☐ Every 7 to 12 months
- ☐ I don't know

Main way of contact (choose 1)

- ☐ Electronically (e.g., email)
- ☐ By telephone
- ☐ In person (face to face)

Reason for contact (choose 1)

- ☐ Short term problem not related to my chronic condition(s)
- ☐ Short term problem related to my chronic condition(s)
- ☐ Chronic condition

Main type of support provided (choose 1)

- ☐ Information and advice
- ☐ Emotional support
- ☐ Practical support
- ☐ Care (e.g., assessment, treatment, prescription)

Other types of support provided

- ☐ Information and advice
- ☐ Emotional support
- ☐ Practical support
- ☐ Care (e.g., assessment, treatment, prescription)

Level of satisfaction with support

- ☐ Very unsatisfied
- ☐ Unsatisfied
- ☐ Satisfied
- ☐ Very satisfied

Roughly how many different psychiatric consultants have you seen in the last 6 months?

m) Surgical consultant

☐ I don't have any contact with this professional → Go to question 9.n)

Frequency of contact (choose 1)

☐ Every day
☐ Once or more a week, but not every day
☐ More than once a month, but not every week
☐ Once a month
☐ Every 1 or 2 months
☐ Every 3 or 4 months
☐ Every 5 or 6 months
☐ Every 7 to 12 months
☐ I don't know

Main way of contact (choose 1)

☐ Electronically (e.g., email)
☐ By telephone
☐ In person (face to face)

Reason for contact (choose 1)

☐ Short term problem not related to my chronic condition(s)
☐ Short term problem related to my chronic condition(s)
☐ Chronic condition

Main type of support provided (choose 1)

☐ Information and advice
☐ Emotional support
☐ Practical support
☐ Care (e.g., assessment, treatment, prescription)

Other types of support provided

☐ Information and advice
☐ Emotional support
☐ Practical support
☐ Care (e.g., assessment, treatment, prescription)

Level of satisfaction with support

☐ Very unsatisfied
☐ Unsatisfied
☐ Satisfied
☐ Very satisfied

Roughly how many different surgical consultants have you seen in the last 6 months?

☐

n) Other hospital doctors

☐ I don't have any contact with this professional → Go to question 9.o)

Frequency of contact (choose 1)

- ☐ Every day
- ☐ Once or more a week, but not every day
- ☐ More than once a month, but not every week
- ☐ Once a month
- ☐ Every 1 or 2 months
- ☐ Every 3 or 4 months
- ☐ Every 5 or 6 months
- ☐ Every 7 to 12 months
- ☐ I don't know

Main way of contact (choose 1)

- ☐ Electronically (e.g., email)
- ☐ By telephone
- ☐ In person (face to face)

Reason for contact (choose 1)

- ☐ Short term problem not related to my chronic condition(s)
- ☐ Short term problem related to my chronic condition(s)
- ☐ Chronic condition

Main type of support provided (choose 1)

- ☐ Information and advice
- ☐ Emotional support
- ☐ Practical support
- ☐ Care (e.g., assessment, treatment, prescription)

Other types of support provided

- ☐ Information and advice
- ☐ Emotional support
- ☐ Practical support
- ☐ Care (e.g., assessment, treatment, prescription)

Level of satisfaction with support

- ☐ Very unsatisfied
- ☐ Unsatisfied
- ☐ Satisfied
- ☐ Very satisfied

Roughly how many different other hospital doctors have you seen in the last 6 months?

o) Others or organisations (please specify:______________________________)

☐ I don't have any contact with this professional → Go to the next question

Frequency of contact (choose 1)

- ☐ Every day
- ☐ Once or more a week, but not every day
- ☐ More than once a month, but not every week
- ☐ Once a month
- ☐ Every 1 or 2 months
- ☐ Every 3 or 4 months
- ☐ Every 5 or 6 months
- ☐ Every 7 to 12 months
- ☐ I don't know

Main way of contact (choose 1)

- ☐ Electronically (e.g., email)
- ☐ By telephone
- ☐ In person (face to face)

Reason for contact (choose 1)

- ☐ Short term problem not related to my chronic condition(s)
- ☐ Short term problem related to my chronic condition(s)
- ☐ Chronic condition

Main type of support provided (choose 1)

- ☐ Information and advice
- ☐ Emotional support
- ☐ Practical support
- ☐ Care (e.g., assessment, treatment, prescription)

Other types of support provided

- ☐ Information and advice
- ☐ Emotional support
- ☐ Practical support
- ☐ Care (e.g., assessment, treatment, prescription)

Level of satisfaction with support

- ☐ Very unsatisfied
- ☐ Unsatisfied
- ☐ Satisfied
- ☐ Very satisfied

Roughly how many different "others" have you seen in the last 6 months?

10. On average, how many different professionals based in the hospital did you see with regard to your 'health' care in the last 6 months?

- ☐ 1
- ☐ 2
- ☐ 3
- ☐ 4
- ☐ 5
- ☐ 6
- ☐ 7
- ☐ 8
- ☐ 9
- ☐ 10
- ☐ More than 10

11. Which people in <u>your daily life</u> do you perceive as being **important** in you care? (Multiple answers are possible and allowed, please select all those who are relevant for you.)

- ☐ Children
- ☐ Family/relatives
- ☐ Friend(s)
- ☐ Neighbour(s)
- ☐ Partner/spouse
- ☐ None of the above
- ☐ Others or organisations (please specify)

12. For each of the below, please indicate the frequency, type and reason for contact with these people as well as the type of support they give you and how well this answers your needs. (Note: we are talking about support regarding your health and social care.)

a) Children

☐ I don't have any contact → Go to question 12.b)

Frequency of contact (choose 1)
☐ Every day
☐ Once or more a week, but not every day
☐ More than once a month, but not every week
☐ Once a month
☐ Every 1 or 2 months
☐ Every 3 or 4 months
☐ Every 5 or 6 months
☐ Every 7 to 12 months
☐ I don't know

Main way of contact (choose 1)
☐ Electronically (e.g., email)
☐ By telephone
☐ In person (face to face)

Main type of support provided (choose 1)
☐ Information and advice
☐ Emotional support
☐ Practical support
☐ Care (e.g., assessment, treatment, prescription)

Other types of support provided
☐ Information and advice
☐ Emotional support
☐ Practical support
☐ Care (e.g., assessment, treatment, prescription)

Level of satisfaction with support
☐ Very unsatisfied
☐ Unsatisfied
☐ Satisfied
☐ Very satisfied

b) Family/relatives

☐ I don't have any contact → Go to question 12.c)

Frequency of contact (choose 1)

- ☐ Every day
- ☐ Once or more a week, but not every day
- ☐ More than once a month, but not every week
- ☐ Once a month
- ☐ Every 1 or 2 months
- ☐ Every 3 or 4 months
- ☐ Every 5 or 6 months
- ☐ Every 7 to 12 months
- ☐ I don't know

Main way of contact (choose 1)

- ☐ Electronically (e.g., email)
- ☐ By telephone
- ☐ In person (face to face)

Main type of support provided (choose 1)

- ☐ Information and advice
- ☐ Emotional support
- ☐ Practical support
- ☐ Care (e.g., assessment, treatment, prescription)

Other types of support provided

- ☐ Information and advice
- ☐ Emotional support
- ☐ Practical support
- ☐ Care (e.g., assessment, treatment, prescription)

Level of satisfaction with support

- ☐ Very unsatisfied
- ☐ Unsatisfied
- ☐ Satisfied
- ☐ Very satisfied

c) Friend(s)

☐ I don't have any contact → Go to question 12.d)

Frequency of contact (choose 1)
☐ Every day
☐ Once or more a week, but not every day
☐ More than once a month, but not every week
☐ Once a month
☐ Every 1 or 2 months
☐ Every 3 or 4 months
☐ Every 5 or 6 months
☐ Every 7 to 12 months
☐ I don't know

Main way of contact (choose 1)
☐ Electronically (e.g., email)
☐ By telephone
☐ In person (face to face)

Main type of support provided (choose 1)
☐ Information and advice
☐ Emotional support
☐ Practical support
☐ Care (e.g., assessment, treatment, prescription)

Other types of support provided
☐ Information and advice
☐ Emotional support
☐ Practical support
☐ Care (e.g., assessment, treatment, prescription)

Level of satisfaction with support
☐ Very unsatisfied
☐ Unsatisfied
☐ Satisfied
☐ Very satisfied

d) Neighbour(s)

☐ I don't have any contact → Go to question 12.e)

Frequency of contact (choose 1)
☐ Every day
☐ Once or more a week, but not every day
☐ More than once a month, but not every week
☐ Once a month
☐ Every 1 or 2 months
☐ Every 3 or 4 months
☐ Every 5 or 6 months
☐ Every 7 to 12 months
☐ I don't know

Main way of contact (choose 1)
☐ Electronically (e.g., email)
☐ By telephone
☐ In person (face to face)

Main type of support provided (choose 1)
☐ Information and advice
☐ Emotional support
☐ Practical support
☐ Care (e.g., assessment, treatment, prescription)

Other types of support provided
☐ Information and advice
☐ Emotional support
☐ Practical support
☐ Care (e.g., assessment, treatment, prescription)

Level of satisfaction with support
☐ Very unsatisfied
☐ Unsatisfied
☐ Satisfied
☐ Very satisfied

e) Partner/spouse

☐ I don't have any contact → Go to question 12.f)

Frequency of contact (choose 1)

- ☐ Every day
- ☐ Once or more a week, but not every day
- ☐ More than once a month, but not every week
- ☐ Once a month
- ☐ Every 1 or 2 months
- ☐ Every 3 or 4 months
- ☐ Every 5 or 6 months
- ☐ Every 7 to 12 months
- ☐ I don't know

Main way of contact (choose 1)

- ☐ Electronically (e.g., email)
- ☐ By telephone
- ☐ In person (face to face)

Main type of support provided (choose 1)

- ☐ Information and advice
- ☐ Emotional support
- ☐ Practical support
- ☐ Care (e.g., assessment, treatment, prescription)

Other types of support provided

- ☐ Information and advice
- ☐ Emotional support
- ☐ Practical support
- ☐ Care (e.g., assessment, treatment, prescription)

Level of satisfaction with support

- ☐ Very unsatisfied
- ☐ Unsatisfied
- ☐ Satisfied
- ☐ Very satisfied

f) Others or organisations (please specify:______________________________)

☐ I don't have any contact → Go to the next question

Frequency of contact (choose 1)
☐ Every day
☐ Once or more a week, but not every day
☐ More than once a month, but not every week
☐ Once a month
☐ Every 1 or 2 months
☐ Every 3 or 4 months
☐ Every 5 or 6 months
☐ Every 7 to 12 months
☐ I don't know

Main way of contact (choose 1)
☐ Electronically (e.g., email)
☐ By telephone
☐ In person (face to face)

Main type of support provided (choose 1)
☐ Information and advice
☐ Emotional support
☐ Practical support
☐ Care (e.g., assessment, treatment, prescription)

Other types of support provided
☐ Information and advice
☐ Emotional support
☐ Practical support
☐ Care (e.g., assessment, treatment, prescription)

Level of satisfaction with support
☐ Very unsatisfied
☐ Unsatisfied
☐ Satisfied
☐ Very satisfied

13. How do you feel about navigating (i.e. finding the right service at the right time) the care system?

- ☐ I find it extremely difficult.
- ☐ I find it difficult.
- ☐ I find it somewhat difficult.
- ☐ I find it somewhat easy.
- ☐ I find it easy.
- ☐ I find it extremely easy.

14. What is the main reason(s) you feel this way?

Background information

In this section you will be asked about yourself and any of the long term health conditions you may have. There are no right or wrong answers.

1. Which of the following long term health conditions have you been diagnosed with? (Multiple answers are possible and allowed, please select all the options that apply to you.)

- ☐ Prefer not to answer
- ☐ Cancer
- ☐ Cardiovascular disease (e.g., heart disease, stroke, high blood pressure)
- ☐ Chronic back pain
- ☐ Chronic bowel disease (e.g., irritable bowel syndrome, Chrohn's disease)
- ☐ Chronic fatigue syndrome
- ☐ Chronic kidney disease (e.g., kidney failure)
- ☐ Chronic neurologic disorder (e.g., Alzheimer, dementia, epilepsy, muscular disease)
- ☐ Chronic respiratory disease (e.g., asthma, chronic obstructive pulmonary disease [COPD])
- ☐ Chronic skin disease (e.g., psoriasis)
- ☐ Diabetes
- ☐ HIV/AIDS
- ☐ Liver disease (e.g., liver cirrhosis)
- ☐ Mental illness (e.g., schizophrenia, bipolar disorder, depression)
- ☐ Musculoskeletal disease (e.g., arthritis, rheumatism, osteoporosis)
- ☐ Thyroid problems
- ☐ Other (please specify:____)

2. How many long term health conditions are you currently diagnosed with? (please write down the total number of long term health conditions)

3. How long ago were you diagnosed with your 'first' long term health condition?

- ☐ Less than 6 months
- ☐ More than 6 months, but less than 1 year
- ☐ 1 year or more, but less than 2 years
- ☐ 2 years or more, but less than 5 years
- ☐ 5 years or more, but less than 10 years
- ☐ 10 years or more
- ☐ I don't know
- ☐ Other (please specify:____)

4. How long since your most recent long term health condition was diagnosed?

- ☐ Less than 6 months
- ☐ More than 6 months, but less than 1 year
- ☐ 1 year or more, but less than 2 years
- ☐ 2 years or more, but less than 5 years
- ☐ 5 years or more, but less than 10 years
- ☐ 10 years or more
- ☐ I don't know
- ☐ Other (please specify:____)

5. Which ethnic group describes your background the best? (please select one group and specify the subgroup).

- ☐ White
  - ☐ English/Welsh/Scottish/Northern Irish/British
  - ☐ Irish
  - ☐ Gypsy or Irish Traveller
  - ☐ White European (e.g., Polish, French)
  - ☐ Other white background (e.g., Australian) – please specify:___________

- ☐ Mixed/Multiple ethnic groups
  - ☐ White and Black Caribbean
  - ☐ White and Black African
  - ☐ White and Asian
  - ☐ Any other mixed/multiple ethnic background – please specify:________

- ☐ Asian/Asian British
  - ☐ Indian
  - ☐ Pakistani
  - ☐ Bangladeshi
  - ☐ Chinese
  - ☐ Any other Asian background – please specify:____________________
- ☐ Black/African/Caribbean/Black British
  - ☐ African
  - ☐ Caribbean
  - ☐ Any other Black/African/Caribbean/Black British group – please specify
- ☐ Other ethnic group
  - ☐ Arab
  - ☐ Any other group – please specify:____________________

6. Where do you currently live in England?

- ☐ Prefer not to answer → *Go to question 10*
- ☐ East Midlands → *Go to question 7*
- ☐ East of England → *Go to question 10*
- ☐ London → *Go to question 10*
- ☐ North East England → *Go to question 10*
- ☐ North West England → *Go to question 10*
- ☐ South East → *Go to question 10*
- ☐ South West → *Go to question 10*
- ☐ West Midlands → *Go to question 10*
- ☐ Yorkshire & Humber → *Go to question 10*
- ☐ Other (please specify:_________________________________)

7. Which region within East Midlands do you currently live in?

- ☐ Prefer not to answer
- ☐ Nottinghamshire
- ☐ Derbyshire
- ☐ Leicestershire
- ☐ Rutland
- ☐ Northamptonshire
- ☐ Lincolnshire

8. Would you like to participate in an interview, about the role different people play with regard to your care, for this study? (Please do not forget to provide us with your contact details below)

☐ Yes ☐ No

9. Are you currently living in or within a 10 mile radius of Lincoln City?

☐ Yes ☐ No

10. Would you like to be contacted by a researcher to talk a little about whether you found this questionnaire difficult or if anything concerns you regarding this questionnaire?

☐ Yes ☐ No

11. How would you like to be contacted? (Please fill out the details for the method of contact you prefer.)

Name:

Address

City/Town

County

Postal Code

Country

Email address

Phone number

You have reached the end of this questionnaire. If you have any other comments about please express them below (this could include comments from the interviewee or problems completing the questionnaire)

Contact details:

Jolien Vos
Graduate Research Assistant
PhD Student
jvos@lincoln.ac.uk
01522 886934

Dr Karen Windle
Reader in Health
Project Supervisor
kwindle@lincoln.ac.uk
01522 886173

Community and Health Research Unit
School of Health and Social Care
University of Lincoln
Brayford Pool
Lincoln, LN6 7TS

Thank you for taking the time to complete this questionnaire

CaHRU
Community and Health Research Unit
