# Supplementary material for: Understanding Care Navigation by Older Adults With Multimorbidity: Mixed-Methods Study Using Social Network and Framework Analyses
Source: JMIR Aging. 2018 Nov 14;1(2):e11054. doi: 10.2196/11054 (PMC6716432; doi:10.2196/11054)
Supplement: Multimedia Appendix 2 [file aging_v1i2e11054_app2.pdf]

INVESTIGATING THE FEASIBILITY AND ACCEPTABILITY REGARDING THE USE OF ICT TO SUPPORT OLDER PEOPLE WITH MULTIMORBIDITY IN THEIR NAVIGATION THROUGH THE CARE SYSTEM.

TOPIC GUIDE (OCTOBER 2015)

INTRODUCTION

The purpose of this interview is to explore your experience on the care network that surrounds you. We are trying to gain an insight into how the care network surrounding older adults with multimorbidity functions. There are no right or wrong answers; the interview is simply about hearing your views on this topic and learning from your experience.

There might be moments during the interview at which we take a look at some of your data from the questionnaire. This can help us build the image of your care network and work with concrete examples.
We will not use your name in any reports of this work and it will not be made known who took part. However, some of the things you say in the interviews might be used to illustrate and support the findings of the research. We will make every effort to make sure that these remain unidentifiable.

Are you happy for this interview to be tape recorded? Only researchers who are part of the team will have access to the recording and you will not be named on the tape.

THE CARE NETWORK

1. **Could you first tell me what you understand by the term 'care network'?**

   Prompts:
   - What does this mean to you?
   - Who supports you regarding your care?
   - Who is involved in your (social and health) care?
   - Who is important to you in your (social and health) care?

2. **How has having a number of health problems or different diagnoses shaped this care network?**

   Prompts:
   - To what extent did it increase the amount of people involved in your care?
   - To what extent did it complicate your life?

ROLES AND RESPONSIBILITIES

3. **Could you tell me something about how and what you do to stay healthy?**

   Prompts:
   - What do you do to remain your well-being?
   - How do you manage your different health conditions?

4. **What do you feel is the role of the people involved in your 'care'?**

   Prompts:

   - E.g., where do you feel your 'role' as a 'patient' stops and the role of the others start?
   - What is the role of friends, relatives, neighbours, etc.?
   - What is the role of social care providers?
   - What is the role of health care professionals?

RELATIONSHIPS AND INTERACTION

5. **How do you feel about these roles?**

   Prompts:
   - To what extent would you like more/less responsibility?
   - To what extent do you think this is in balance?
   - To what extent do you feel 'in control' of/listened to for your own care?

6. **Tell me something more about how the people that support you, interact with you?**

   Prompts:
   - How do they communicate with you?
   - If so, how? If not, what do you think about this?

7. **Tell me something more about how they work with/or alongside each other?**

   Prompts:
   - Do people know about the other people involved in your care?
   - Do they communicate with each other?
   - If so, how? If not, what do you think about this?

8. **What things do you think work well across your care network?**

   Prompts:
   - Who/What are you satisfied with and why?
   - Who/What are you less satisfied with and why?

9. **What could be improved across your care network?**

   Prompts:
   - For instance, what is the communication like between your GP and specialists you see in the hospital?
   - How could this be improved for you?

NAVIGATING THE CARE SYSTEM/NETWORK

10. **How do you manage your care network practically when having a number of health problems or diagnoses?**

    Prompts:
    - For instance, some people see many care providers and have a busy schedule of appointments, how is this for you?
    - How do you know who to contact, go to, with which concern?

11. **What makes you finding your way through the health and social care support that you need, easier?**

Prompts:
- Which information do you rely on to know who to go to?
- Who informs you about the services available to you?
- Which people do you rely on to know who to go to?

12. **What makes finding your way through the health and social care support that you need, difficult?**

Prompts:
- For instance, some people find it difficult to juggle the different sites at which they need to be to get the help they need. How is this for you?
- What could be improved?
- What would be helpful for you to find your way through the system?

TECHNOLOGY AND HOW IT COULD SUPPORT

In this section the participant might be provided with some paper materials and examples that are possible to develop on an electronic platform.

13. **Can you tell me something about technology (e.g., mobile phone, computer) you currently use?**

Prompts:
- For what do you generally use these?
- If they don't use: What do you think about technology?
- How do you use it in relation to health and social care (e.g., booking appointments)?

14. **In an ideal world, what would the care system look like for you?**

Prompts:
- How would it function ideally?
- What things are in place in this world to help you?

ROUND-UP

Thank you very much for your time and your help with this study! Are there any further comments you would like to make that you don't think we picked up through the discussion?
